# Factors that potentially influence successful weight loss for adults with intellectual disabilities: A qualitative comparison

**DOI:** 10.1177/1744629520931681

**Published:** 2020-06-24

**Authors:** Laura J Skelly, Philomena P Smyth, Mark P Donnelly, Julian C Leslie, Geraldine Leader, Liz Simpson, Claire McDowell

**Affiliations:** Ulster University, UK; Ulster University, UK; National University of Ireland Galway, Ireland; Ulster University, UK; Ulster University, UK; National University of Ireland Galway, Ireland; Ulster University, UK; Ulster University, UK

**Keywords:** barriers, facilitators, focus groups, intellectual disabilities, weight loss

## Abstract

**Background:**

People with intellectual disabilities are more at risk of obesity than the general population. Emerging literature indicates that multicomponent interventions are most effective, however, individual results are variable and little research exists as to why this is the case.

**Methods:**

Focus groups were conducted to explore lived experiences between two groups of adults with intellectual disabilities; an overweight group (*n* = 6) and a group identified as successful in losing weight (*n* = 6). Similarities and differences were explored across four domains. Transcripts were produced and analysed using Theoretical Thematic Analysis.

**Results:**

Similarities included service centre supports, basic food knowledge and issues restricting independence. The successful weight loss group had also internalised health messages, engaged with external reinforcement programmes, responded to positive feedback and demonstrated healthier dietary habits.

**Conclusion:**

Weight management interventions would benefit from understanding the influence that internalisation of health messages, effective reinforcement systems and positive feedback can have on supporting the adoption of healthier habits.

## Introduction

The growth of overweight within society over the last four decades constitutes a worldwide epidemic (Swinburn et al., 2011), with approximately 1.9 billion overweight adults worldwide, of whom around 650 million are obese (World Health Organisation, 2019). Furthermore, adults with intellectually disabilities are particularly vulnerable to becoming overweight. Recent prevalence rates quoted in the literature for overweight adults with intellectual disabilities range from 52% to 67.2%, compared to figures for the general population which range from 43.4% to 61.3%. While the difference in prevalence between those with intellectual disability and the general population may not appear to be great, the rate of obesity, categorised as a body mass index (BMI) of greater than 30 kg/m^2^, is far greater for the intellectually disabled population. Obesity prevalence ranges from 17.6% to 38.3% for the intellectually disabled population compared to 11.8% to 28% for the general population (Hsieh et al., 2014; Koritsas and Iacono, 2016; Mikulovic et al., 2014; San et al., 2016).

People with intellectual disabilities are also less likely to access healthcare facilities or initiatives (Krahn et al., 2019), placing them at higher risk of diabetes type 2, cardiovascular disease and certain cancers, presenting further concerns for individuals and national healthcare providers (Wang et al., 2011). However, until governments make dramatic policy changes to tackle the causes of obesity at a population level (Swinburn et al., 2011), overweight individuals must continue to find ways to moderate the drivers of obesity at a personal level for now.

Current guidelines for effective interventions among the general population recommend multicomponent interventions (MCIs) which combine a dietary component, increased physical activity component and behavioural change strategies (National Institute for Health and Care Excellence, 2014). There are no such guidelines available for the intellectually disabled population to date, however, several reviews are present within the literature which aim to guide the design of future interventions. In a review by Hamilton et al. (2007), interventions were grouped according to the components they comprised of. The review concluded that behavioural strategies, physical exercise, dietary knowledge and carer assistance were all effective in producing weight losses in the short term. However, there was no comparison between components to identify the most effective component or combination of components. Spanos et al. (2013) provided such a comparison in their review and concluded that MCIs with a dietary component, increased physical activity component and behavioural change strategies were the most effective for the adult intellectually disabled population, echoing the guidelines for the general population. In their review of randomised control trials for MCIs with adults with intellectual disabilities, Harris et al. (2018) found that only the MCIs that included an energy-deficient diet as part of the intervention produced significant weight losses. Therefore, as in the general population, MCIs that incorporate a calorie deficit as their dietary component, an increase in physical activity and proven behaviour change strategies, appear to be the best choice for adults with intellectual disabilities. While these types of intervention show significant group effects, individual success remains variable across participants and few data exist to analyse longer term outcomes.

Identifying barriers and facilitators for adults with intellectual disabilities trying to achieve a healthier lifestyle may shed light on possible individual differences. At present, the literature reports common barriers and facilitators to exercise engagement (Bodde and Seo, 2009; Frey et al., 2005; Messent et al., 1999; Temple and Walkley, 2007), and some studies have touched on barriers and facilitators to healthy eating (Kuijken et al., 2016). However, there are not many studies available that analyse differences between individuals to help explain the variability in weight loss between participants. One factor noted by Heller et al. (2002) was the correlation between the success or failure of participants to exercise, and caregivers’ personal beliefs on whether exercise would benefit the person. Temple (2007) also studied differences in physical activity by comparing perceived barriers or activity preferences between groups of active and sedentary adults with intellectual disabilities. She found that participants demonstrating lower step counts also listed more barriers. These studies suggest possible reasons for variance across this population, however, there remains a lack of direct evidence of differences in lifestyles between healthy weight and overweight adults with intellectual disabilities. Further research is warranted to understand factors that influence lifestyle differences between adults with intellectual disabilities who achieve weight loss and those who do not.

This study aimed to explore the similarities and differences of two groups that differed in weight status and healthy lifestyle behaviours, across four domains of interest in relation to healthy eating and exercising: facilitators; barriers; knowledge base; and current habits.

## Methods

### Rationale

Researchers have actively involved people with intellectual disabilities in the research process, and the inclusion of adults with intellectual disabilities in research that proposes to serve their needs has now become policy driven (Gilbert, 2004). Phenomenology was chosen as the best fitting methodology for this study as its theoretical underpinning allows for discussion to develop freely in relation to the thoughts, feelings, opinions and experiences of adults with intellectual disabilities in relation to diet and exercise (Guest et al., 2012). Focus groups were chosen as the specific method to gain insight, as these have proven effective in eliciting information from adults with intellectual disabilities on matters that affect their lives (Gates and Waight, 2007; Kaehne and O’Connell, 2010). It was expected that the social interaction aspect of focus groups would provide insight into shared and idiosyncratic lived experiences of these adults in relation to exercise and healthy eating. For focus groups to be successful, participants should have some commonality in relation to the topic (Asbury, 1995).

### Participants

Group 1 participants (*n* = 6) were recruited as part of a larger study relating to weight management for adults with intellectual disabilities. Participants were invited to join the larger study via the service manager if they satisfied the following criteria: aged 18 or over;, mild or moderate intellectual disability; and BMI >25 kg/m^2^. Group 1’s focus group ran prior to any health promotion information or weight management intervention being conducted. Group 2 participants were a convenience sample recruited from the same service centre specifically to support commonality in the opportunities available to them with respect to healthy eating and exercise, and to highlight potential differences between individuals that achieve weight loss and those that do not. Group 2 participants (*n* = 6) were invited to join this study via the service manager if they satisfied the following criteria: aged 18 or over; mild or moderate intellectual disability; and had achieved and maintained a substantial amount of weight loss over the previous 12 months (‘substantial amount of weight’ was a subjective measure determined by the service manager). Participants were excluded from both groups if they showed any challenging behaviours or mental health issues that would unduly jeopardise participation in the study. As indicated in Table 1, the groups were similar except for marked differences in their BMI scores prior to the study.

**Table 1.** Participant demographics by group.

Demographic	Group 1	Group 2
Number of participants	6	6
Males	2	2
Females	4	4
Mean age	49	45
Age range	38–59	25–73
Number of participants in each living situation		
With family	3	5
Supported accommodation	1	1
Own home	1	0
Number of participants in each BMI category		
Healthy weight (18–24.9 kg/m^2^)	0	2
Overweight (25–29.9 kg/m^2^)	0	3
Obese 1 (30–34.9 kg/m^2^)	2	1
Obese 2 (35–39.9 kg/m^2^)	3	0
Obese 3 (>40 kg/m^2^)	1	0

BMI: body mass index.

### Procedure

#### Setting and structure

Both focus groups were conducted on the same day within the service centre in a designated room; group 1 in the morning and group 2 in the afternoon. Each session lasted approximately 90 minutes with a 15-minute break. Two of the authors attended, one as lead moderator and the other as assistant moderator. The lead moderator was responsible for delivery of questions, encouraging conversation between participants, and realigning the conversation towards the study goals where necessary. The assistant moderator was responsible for note taking and organisation of visual supports. Each participant could bring a support person if they wished, however, all attended independently. The discussions were audio-recorded to allow transcripts to be developed and analysed.

#### Anthropometric measures

Participants’ heights and weights were collected by two researchers at the end of each focus group discussion. Participants were measured wearing a t-shirt, light trousers and no socks or shoes. Measures were conducted by one of the researchers while the second researcher observed, and agreement was reached. A stadiometer, Charder HM200P, was used to measure height in feet and inches to the nearest 0.5 inch. The height of each participant was then programmed into the Smart Weigh SW-SBS500 Digital Body Fat Scale to allow automatic calculation of BMI, and participants were instructed to stand on the scale barefoot until both weight in lbs, to the nearest 0.1 lbs, % body fat and BMI were recorded.

### Materials

The framework of questions devised followed the five-question framework outlined by Kruegar and Casey (2015). This framework creates a logically sequenced series of open-ended questions where the beginning questions are more general, then subsequent questions become more focused to elicit more specific information. Table 2 details the questions devised by two researchers who acted as facilitator and assistant facilitator for both focus groups. The introduction and transition questions were influenced by the Transtheoretical Model to establish what stage of change each participant may have been operating at, (1) Pre-contemplation, (2) Contemplation, (3) Action, (4) Maintenance or (5) Process Complete/Relapse (Marks et al., 2018), and therefore provide insight into the influences affecting attitudes and opinions provided by participants during the key questions. Additionally, these questions were designed to establish whether participants understood the need to eat healthy foods and exercise in order to lose weight. The key questions were aimed at eliciting whether participants could identify factors present in their own lives that aided or hampered healthy choices. These questions were influenced by current research with the adult intellectually disabled population which examines levels of autonomy, opportunity and ability to eat healthy diets and exercise (Bodde and Seo, 2009; Frey et al., 2005; Kuijken et al., 2016; Messent et al., 1999; Temple and Walkley, 2007). Information relating to current knowledge and habits was also sought.

**Table 2.** Questions and structure for focus groups.

Question type	Details
Opening	Can you tell us your name and something about yourself?
Introduction	Can you tell us about your experiences of managing your weight so it doesn’t get too big or too high?
Transition	How long have you been aware that you need to lose weight?
Key 1	How do you plan and make your meals? (supporting visuals used)
Key 2	Do you do your own shopping or does someone help you?
Key 3	What foods do you like or dislike? (supporting visuals used)
Key 4	How do you fill your free time in the evenings and at weekends?
Key 5	What kinds of exercise do you do each week? (supporting visuals used)
Key 6	What activities do you not like doing? (supporting visuals used)
Conclusion	Summary of topics discussed. ‘Of all the things we have talked about which ones are really important?’ ‘Is there anything else about health or losing weight that you want to talk about?’

### Consent and ethical approval

The study was approved by a University Ethics Committee and was conducted in full accordance with World Medical Association Declaration of Helsinki (2002). Particular attention was given to issues of informed, voluntary consent by participants, and in each case, ability to give consent was corroborated by a caregiver who knew them well.

### Data analysis

Transcripts of the audio recordings were produced and subsequently coded using Theoretical Thematic Analysis (Braun and Clarke, 2006). The initial coding was conducted by the first author before being reviewed by a second author who co-facilitated the focus groups (PS). Queries in the coding were discussed between the two researchers and agreement reached in all cases. Common themes were developed from the transcripts with respect to the four domains of interest: (1) facilitators; (2) barriers; (3) knowledge base; and (4) current habits. The themes for each group were then compared to find possible differences between adults with intellectual disabilities that achieve and maintain weight loss, against adults with intellectual disabilities that are overweight.

## Results

**Figure 1.**
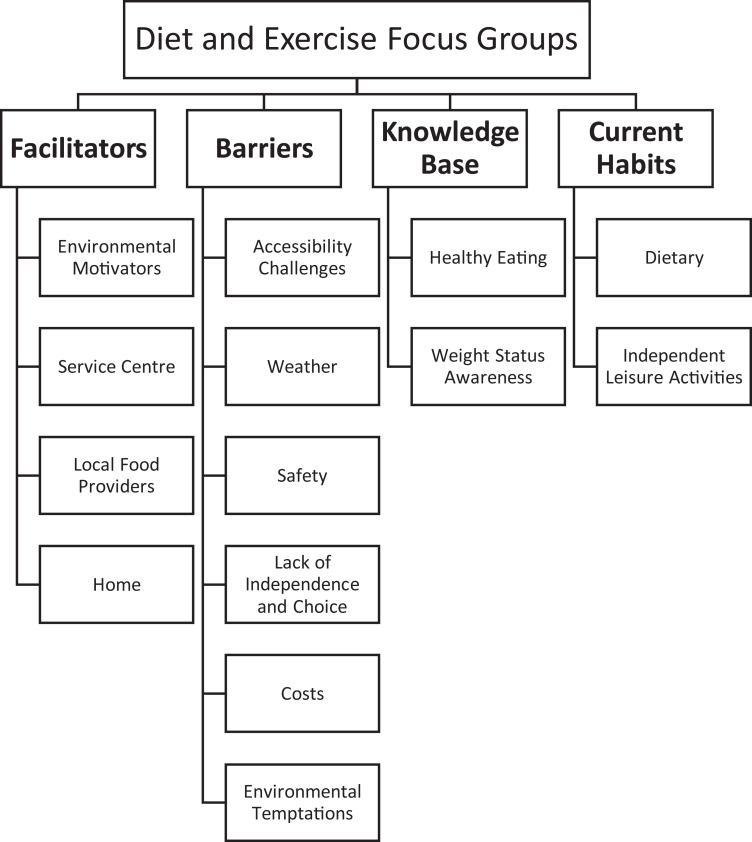
Thematic map of themes developed under each domain of interest.

Participants interacted well with each other in both groups and all contributed to the overall conversation. A variety of themes emerged under each of the four domains of interest, all of which are shown in Figure 1. Facilitator themes consisted of people, places or events that participants identified as aiding them to engage in healthy eating or exercise. Barrier themes related to comments made about any aspect of their lives that prevented them from engaging in healthy eating or exercise. Demonstrations of knowledge relating to weight, health, foods or exercise by participants were captured under the domain of knowledge base, and the current habits theme included any reference made to current eating or exercising habits.

The total number of quotations coded from each focus group was 85 for group 1 and 101 for group 2, with the number of quotations used to develop each of the themes for each of the groups detailed in Table 3. A comparison between the thematic analyses of the two groups identified similarities and differences in these themes for each domain.

**Table 3.** Number of quotes used to generate each theme by group.

Domain	Theme	Group 1	Group 2
Facilitators	Environmental motivators	4	23
	Service Centre	3	16
	Local food providers	0	3
	Home	2	2
	Total	9	44
Barriers	Accessibility challenges	0	5
	Weather	5	2
	Safety	2	4
	Lack of independence and choice	5	2
	Costs	3	3
	Environmental temptations	3	2
	Total	18	18
Knowledge Base	Healthy eating	3	11
	Weight status awareness	5	4
	Total	8	15
Current Habits	Dietary	26	6
	Independent leisure activities	24	18
	Total	50	24
Total number of quotations		85	101

### Facilitator themes

Table 4 details emergent themes and sub-themes for each group with respect to factors that facilitate healthy lifestyle choices.

**Table 4.** Facilitator themes and sub-themes by group.

Domain: Facilitators			
Themes	Sub-themes	Group 1	Group 2
Environmental motivators	External reinforcement programmes		√
	Positive feedback		√
	Mental health	√	√
	Positive influence of role models	√	√
	Awareness of overweight status	√	√
Service Centre	Facilitating exercise engagement	√	√
	Facilitating healthy eating		√
	Health promotion messages		√
Local food providers	Solutions for healthy eating		√
Home	Facilitating exercise engagement	√	√

#### Environmental motivators

Both groups emphasised the benefits of exercise on *mental health* in their own lives. The *positive influence of role models* with regard to eating habits and physical appearance, and an *awareness of their overweight status* were also noted as powerful motivators towards a healthier lifestyle for both groups.

Group 2: PARTICIPANT 2.0   ‘I walk, if I don’t I get annoyed. Otherwise I’d be anxious, so I go for my walk’.

Group 1: PARTICIPANT 1.0   ‘If you see someone healthy eating and you’d have to do it too’.

Group 2: PARTICIPANT 2.4   ‘Well what was happening to me going out to buy clothes and see somebody there in a smaller size’.

Two further motivating sub-themes that emerged solely for group 2 were *external reinforcement programmes* run by the Service Centre, and *positive feedback* from others with respect to weight lost. Group contingency initiatives run by the Service Centre that concentrated on providing reinforcement for engaging in healthy eating or exercise were referred to many times during group 2’s conversation. Additionally, the reaction and positive feedback of others in relation to weight lost was stated as an influential motivator to maintain lifestyle changes by group 2.

Group 2: PARTICIPANT 2.4    ‘If we go out walking we have these tickets and we get one. If we go out walking, relaxation, eat fruit and veg, and what’s the other one?’

Group 2: PARTICIPANT 2.1   ‘and now people are saying to me I’m losing weight and I’m so happy about that’.

#### Service Centre

*Exercising opportunities* provided by the service centre were spoken about by both groups, with group 2 providing more volume of discussion and more about the variety of exercises available that they take part in. Both groups are provided with the same opportunities for exercising, however, group 2 availed of these opportunities more than group 1 and therefore added more to the discussion for this. A specific exercise group existed for over 50s, however, both groups had three participants who satisfied this criterion, so the opportunity to engage with this would have been the same for both groups.

Group 1: PARTICIPANT 1.1   ‘we do exercise in the centre. I walked around [park name] yesterday, we do that twice a week, and we do exercise here as well as in the aging opportunity room’.

Group 2: PARTICIPANT 2.5   ‘We do circuits here every morning’.

The service centre was also credited by group 2 for *facilitating healthy eating* by providing healthy meal alternatives for anyone wishing to avail of them. Health risks associated with overweight were highlighted in *health promotion messages* by the service manager and served as a motivator to exercise for group 2.

Group 2: PARTICIPANT 2.5   ‘[the dinners delivered to the centre] they are beautiful dinners. They are healthy’.

Group 2: PARTICIPANT 2.1    ‘[Service manager] said you’s will get heart attacks and stroke and everybody has thought about that and I think it’s the walking and the running that, I think the staff should get praise for that’.

#### Local food providers

Group 2 were the only group that provided possible *solutions for healthy eating* by stating local healthy food providers that were not too expensive.

Group 2: PARTICIPANT 2.1   ‘if you went to the college you could get healthy options, better value’.

#### Home

Group 1 noted that the presence of pet dogs *facilitated exercise* in the form of walking in the evenings and at weekends, and group 2 reported that family members encouraged exercise outside of service centre hours.

Group 1: PARTICIPANT 1.0   ‘I have a dog and I walk him every day’.

Group 2: PARTICIPANT 2.4   ‘exercise bike, I do it with my sister’.

### Barrier themes

Table 5 details emergent themes and sub-themes for each group with respect to barriers towards healthy lifestyle choices.

**Table 5.** Barrier themes and sub-themes by group.

Domain: Barriers			
Themes	Sub-themes	Group 1	Group 2
Accessibility challenges	Accessing community exercise facilities		√
Weather	Poor weather conditions	√	√
Safety	Road safety	√	√
	Personal safety	√	√
Lack of independence and choice	Independent opportunities	√	√
Environmental temptations	Difficulty in avoiding temptations	√	√
Cost	Expense of healthy food	√	√
	Expense of community exercise facilities		√

#### Accessibility challenges

Group 2 were the only group to discuss accessibility challenges involved in using *community exercise facilities*. The physical act of getting there posed numerous difficulties such as the distance of travel, the terrain around the facility, a lack of transport to the facility and the cost of transport to the facility if needed.

Group 2: PARTICIPANT 2.1   ‘I can’t nearly walk right and the gym, I know my friend could walk up to it, but I can’t and it’s €5 for a taxi up’.

#### Weather and safety

All participants agreed that *poor weather conditions*, *road safety* and *personal safety* concerns were barriers to exercising as they reduced both motivation and ability to exercise independently.

Group 1: PARTICIPANT 1.1   ‘You can’t go out when it’s raining’.

Group 2: PARTICIPANT 2.3   ‘once there is nobody around you would feel a bit nervous, no I wouldn’t go out when it’s dark’.

#### Lack of independence and choice

Most participants in both groups lived at home with family. For these participants, family-based shopping and cooking were raised as barriers to healthy eating as participants often felt they *lacked independence and choice* around the foods purchased and essentially then the foods they ate.

Group 1: PARTICIPANT 1.2   ‘Your mother [decides on the food you eat]’.

Group 2: PARTICIPANT 2.2   ‘my mam does the shopping sometimes but if there is anything I need like, or if I’m in the house on my own I would see how much money I have and I’d go down the town and get what I need’.

#### Cost

Since healthy foods were thought of as more expensive by both groups, *cost of healthy food* became a noted barrier by both groups. Group 2 also noted the expense involved in community exercise facilities.

Group 1: PARTICIPANT 1.3   ‘healthy food is dearer’.

Group 2: PARTICIPANT 2.2   ‘[It would be easier to exercise if] there is somewhere you can go that is free and you don’t need to pay’.

#### Environmental temptations

When faced with independent food choices in the community, both groups admitted to *struggling to avoid temptations* in cafes or shops.

Group 1: PARTICIPANT 1.1   ‘Or you go in to [a café] to get tea that’s the hard time. Will I have something with that tea or will I have tea on its own?’

Group 2: PARTICIPANT 2.2   ‘when the coffee shop used to be open over there, there was only, well there was healthy options like salad but in the other, over in the hot there was just temptation. Do I go to this or do I go to that? What do I do?’

### Knowledge Base themes

Table 6 details emergent themes and sub-themes for each group with respect to knowledge base around health.

**Table 6.** Knowledge Base themes and sub-themes by group.

Domain: Knowledge Base			
Themes	Sub-themes	Group 1	Group 2
Healthy eating	Types of healthy foods	√	√
	Types of unhealthy foods	√	√
	Portion size		√
Weight status	Awareness of being overweight	√	√
	Association between overweight and reduced health		√

#### Healthy eating

Both groups demonstrated knowledge in relation to commonly known *healthy and unhealthy foods*, but group 2 were the only group to mention the importance of *small portions*.

Group 1: PARTICIPANT 1.3   ‘And eat lots of fruit’.

Group 2: PARTICIPANT 2.2   ‘[eat] small portions’.

#### Weight status awareness

Overweight participants in both groups *acknowledged that they were overweight* and made references to the past when they hadn’t been overweight. Some participants also provided possible reasons for becoming overweight.

Group 1: PARTICIPANT 1.1   ‘I was always thin when I was growing up, until lately. Until I started to eat sweet things until they come out of my eyes. And I put on weight’.

The link between being *overweight and reduced health outcomes* was only referred to during group 2’s discussion.

Group 2: PARTICIPANT 2.2   ‘[when I started putting on weight] I would be all out of breath whenever I walked up the steps or run really fast’.

### Current Habits themes

Table 7 details emergent themes and sub-themes for each group with respect to current eating and leisure habits engaged in.

**Table 7.** Current Habits themes and sub-themes by group.

Domain: Current Habits			
Themes	Sub-themes	Group 1	Group 2
Dietary	Healthy habits	√	√
	Unhealthy habits	√	√
Independent leisure activities	Physical activities	√	√
	Sedentary activities	√	√

#### Dietary

Both groups spoke about *healthy eating habits* by claiming to drink more water, eat more fruit and vegetables and eat smaller amounts of food. Participants from both groups confessed to consuming the occasional treat in coffee shops as *unhealthy eating habits*.

Group 1: PARTICIPANT 1.3   ‘I drank 6 beakers of water yesterday to get my weight off’.

Group 2: PARTICIPANT 2.5   ‘I’m very fond of a lot of fruit, and I buy a lot of fruit’.

Group 1: PARTICIPANT 1.4    ‘I don’t eat too much at home either. I eat my dinner here every day and that does me’.

Group 1: PARTICIPANT 1.4   ‘I treat myself every Friday, once a week to a small cup of cappuccino coffee in the [café name]’.

Numerous additional *unhealthy eating habits* were stated by group 1. Discussions relating to regularly eating treats, drinking fizzy drinks and alcohol and consuming takeaway foods occurred with great frequency throughout group 1’s conversation. These habits were not referred to in group 2’s conversation.

Group 1: PARTICIPANT 1.0   ‘I only have coke’.

Group 1: PARTICIPANT 1.2   ‘I do [drink alcohol], I do, I do. I’d have three, aye, that’s the whole’.

Group 1: PARTICIPANT 1.3    ‘my brother gets Chinese on a Saturday, I love Chinese yeah, he gets Chinese for the two of us and we share’.

#### Independent leisure activities

Independent leisure activities were similar for both groups with walking being the main form of *physical activity* and watching TV being the main *sedentary activity* for both groups. Both groups also helped with household chores which provided another form of *physical activity*.

Group 2: PARTICIPANT 2.5   ‘I walk at home and I walk here as well’.

Group 1: PARTICIPANT 1.1   ‘watch television, sitting down’.

Group 1: PARTICIPANT 1.5   ‘I just clean my house, water my flowers then’.

## Discussion

The comparison of sub-themes between groups showed numerous similarities while also highlighting some important differences. Similar themes emerged for both groups under all four domains. However, knowledge of healthy options, increased practices of healthy behaviours, and higher levels of motivation towards weight loss differed, with group 2 demonstrating wider awareness of these factors.

*Facilitators*: All the overweight adults in this study were aware of being overweight and quoted their weight status as a motivator to lose weight. However, weight status was spoken of in terms of actual weight (stones and lbs) with no comprehension of how this relates to a healthy weight or to BMI category. Without the ability to compare actual weight to a specific target it was impossible for participants to understand the amount of weight they needed to lose. Providing a target weight that equates to a 10% weight loss would be advantageous as a starting point for obese adults with intellectual disabilities as a 10% weight loss has been shown to produce significant health gains (Mertens and Van Gaal, 2000). In fact, many of the studies available suggest that self-perceptions of weight status are distorted in adults with intellectual disabilities, with a tendency towards underestimation, particularly in females (Ayaso-Maneiro et al., 2014; Eden and Randle-Phillips, 2017). A more positive perception may have advantages for self-esteem but may reduce the level of motivation required to achieve and sustain weight loss. While body dissatisfaction is a driver for weight loss (Johnson and Wardle, 2005; Stice and Shaw, 2002), the prevalence of overweight is so high (World Health Organisation, 2019) that it is unlikely that weight status alone provides enough influence for sustained weight loss. Perceptions of others, however, may influence weight loss, since both groups stated being motivated to lose weight after observing other people’s healthy behaviours, including the positive results of weight loss. These behavioural processes are social observational learning (Chance, 2014) and vicarious reinforcement (Cooper et al., 2014). Therefore, it may be important to focus on providing positive peer role models in the environments of adults with intellectual disabilities.

Opportunities to engage in exercise, long established as beneficial for weight management and mental health (Callaghan, 2004; Jones et al., 2007), must also be readily available (Mahy et al., 2010; Temple and Walkley, 2007). Both groups noted that home and service centres provided them with these opportunities and that the benefits to mental health encouraged continued exercise. However, providing opportunities does not guarantee engagement. A major difference between the two groups was that group 2 discussed engaging in a higher frequency and variety of regular exercise. Therefore, to ensure increased uptake of exercise for adults with intellectual disabilities, we should explore further how to support families and service centres in promoting exercise opportunities available and tailoring these to service users’ needs and interests.

There was a larger volume of environmental motivators that facilitate healthier lifestyles for group 2 (see Table 3). Two unique examples of environmental motivators provided by group 2 were ‘external reinforcement programmes’ and ‘positive feedback’. At the heart of both is positive reinforcement, a proven behavioural technique used to affect behaviour change (Cooper et al., 2014; Leslie and O’Reilly, 2003). Group 2 emphasised these influences motivating them to lose weight and maintain weight loss. In contrast, for group 1, it may be that unhealthy foods and sedentary lifestyles hold more reinforcing value to them than the external reinforcement programmes and positive feedback provided for more healthy choices. Positive reinforcement has been used successfully in many weight loss interventions with the intellectually disabled population (Bazzano et al., 2009; Fox et al., 1984; Martinez-Zaragoza et al., 2016; Sailer et al., 2006; Saunders et al., 2011). However, the incentives provided in those studies were predetermined for the group and did not account for individual preferences and motivations, which may account for the variability in individual success. Reinforcement functions most effectively when individual preferences are considered (Cooper et al., 2014), therefore, promoting the implementation of individualised reinforcement options that can compete with unhealthy lifestyle choices is something that should be explored for individuals prior to any weight loss intervention. The practicalities and costs of providing this level of service are, however, fraught with difficulties and rely not only on funding but on high levels of commitment from family members and staff who support the adults with intellectual disabilities.

Health promotion interventions are common within the literature for weight loss with adults with intellectual disabilities, but these have had mixed impact (Geller and Crowley, 2009; Mann et al., 2006; Marshall et al., 2003). By citing health risks associated with being overweight and discussing ways to source healthy meal alternatives, group 2 demonstrated internalisation of health promotion messages delivered by staff. The impact that this internalisation has had on weight loss for this group is hard to quantify, however, it may be one aspect facilitating the process. In a study promoting exercise engagement for middle-aged women, Lenneis and Pfister (2017) credited internalisation of government health messages, relating to the health benefits of exercise for middle-aged females, as a catalyst for exercise engagement. The onset of middle age and the associated health risks prompted the women to take part in the intervention. A similar effect is noted by McDermott (2011) and Dallaire et al. (2012) in relation to health behaviours, demonstrating that knowledge of health risks associated with poor lifestyle choices can increase physical exercise and alter dietary habits. While health promotion interventions are common in the weight loss literature for adults with intellectual disabilities (Bergstrom et al., 2013; Chapman et al., 2005; Ewing et al., 2004; Geller and Crowley, 2009; Mann et al., 2006; Marshall et al., 2003; Pett et al., 2013), the main outcome measure is either anthropometric change or improvement in health behaviours. It would be useful to measure the level of internalisation of health messages for each individual and compare this measure to the weight loss achieved to evaluate individual differences and determine the effect of internalisation. At present though, we note that internalisation of health promotion messages may add value and reduce variability if included in the overall framework of an MCI for weight loss.

*Barriers*: Many of the barriers to successful weight loss were mentioned by both groups, including lack of support from others, lack of choice in accessing healthy foods, perceived high costs associated with healthy eating and exercise options, poor weather conditions, road safety, personal safety and the difficulty of avoiding temptations in the environment. Most of these barriers have been found in other studies (Bodde and Seo, 2009; Frey et al., 2005; Messent et al., 1999; Temple and Walkley, 2007). The main difference between the groups involved the difficulties in accessing community exercise facilities and the expense it incurs. Because very few adults with intellectual disabilities have their own method of transport, accessing community facilities can be challenging. It is possible that group 2 has more insight into these challenges due to participants engaging in or seeking exercise opportunities from sources other than those provided by the service centre, a barrier unique to active adults with intellectual disabilities that was also noted by Temple (2007). This may be an important difference between groups that relates to higher levels of internal motivation for exercise and therefore increased health. While both groups discussed the expense of eating healthy foods, another difference arose between the groups when group 2 added potential solutions by demonstrating ways to problem-solve around additional expense within their own environment.

*Knowledge Base*: Kuijken et al. (2016) demonstrated that most adults with a mild or moderate intellectual disability have grasped the basic themes of healthy living. This study also found that both groups were able to identify healthy and unhealthy foods and knew the importance of drinking water. However, Golden and Hatcher (1997) found that knowledge alone does not predict successful weight loss. Only group 2 discussed the link between being overweight and reduced health, and the effect of portion size on weight. Wansink et al. (2005) discovered that larger portions lead to the consumption of more food. Portion sizes appear to be growing and exceeding recommendations both at home and in the food industry (Condrasky et al., 2012; Kairey et al., 2018). As larger portion sizes become the norm, the quantification of how much should be eaten becomes more difficult for all of us and particularly for adults with intellectual disabilities. Knowing that eating less aids weight loss is a step in the right direction, but it would be interesting to see whether actual portion sizes are smaller for adults with intellectual disabilities that manage weight loss, and how close to the recommended portion sizes they are. Portion size education may be a useful addition to any weight loss intervention for adults with intellectual disabilities and those who support them.

*Current Habits*: Both groups reported eating fruit and vegetables, drinking lots of water and having the occasional treat when they were out. Studies that have tried to document actual food intake for adults with intellectual disabilities have found this to be a challenging task, with most reporting a lack of fruit and vegetables, and a non-balanced diet, biased towards high energy dense foods (Melville et al., 2007). Both groups in this study had a mixed degree of control over their food choices, but the most notable difference in current habits related to group 1 participants who indicated eating treats in the evenings, regularly drinking alcohol or fizzy drinks and regularly eating takeaway foods. With respect to leisure habits, both groups spoke about engaging in similar types of physical (walking) and sedentary (watching TV) activities. It is possible that group 2 may engage in more exercise as a leisure activity since they appear more motivated, though, without an actual measure this is not possible to ascertain.

## Limitations

This study involved a small convenience sample of participants limiting generalisability of the results. Nonetheless, the main findings of the study warrant future exploration on a larger scale. The recruitment of group 2 participants was based on subjective weight losses identified by the service manager. While this lacked rigour, significant weight loss is difficult to achieve for this population, and so gaining insight into the perspectives of those who have achieved noticeable weight loss is worth examining. Lastly, since this sample involved adults with a mild or moderate intellectual disability, the results may not be applicable to those with a severe or profound intellectual disability. However, since adults with a mild or moderate intellectual disability are the most at risk for obesity, pinpointing factors that aid weight loss for this population is paramount.

## Conclusion

To conclude, the two groups encountered many similar experiences across the four domains, in line with previous findings (Bodde and Seo, 2009; Frey et al., 2005; Kuijken et al., 2016; Mahy et al., 2010; Messent et al., 1999; Temple and Walkley, 2007). The influence of family support was noted by both groups as important, particularly in relation to food choices. Carer support has been shown to affect individual outcomes (Fox et al., 1985; Hamilton et al., 2007; Zoppo and Asteria, 2008) and should therefore be noted as a factor that influences each individual’s achievement of weight loss. However, several differences between groups that may strongly influence weight loss were also identified and add to the available literature. These were internalisation of *health promotion messages* which make the link between overweight and reduced health, motivated by effective *external reinforcement programmes* and *positive feedback*, and the presence of *healthier dietary habits* that lack regular consumption of treats, alcohol, fizzy drinks and takeaway foods. Ensuring internalisation of health messages and increasing knowledge around the health value of food would be a worthwhile addition to any weight loss intervention. Providing suggestions of healthy substitutes or alternatives to treats, alcohol, fizzy drinks and takeaway foods, while supporting adoption of these new habits through effective and individualised reinforcement systems and positive feedback appears to be paramount to successful and sustainable weight loss for adults with intellectual disabilities.
